# Supplementary material for: Humoral Immune Responses in Patients with Severe COVID-19: A Comparative Pilot Study between Individuals Infected by SARS-CoV-2 during the Wild-Type and the Delta Periods
Source: Microorganisms. 2023 Sep 20;11(9):2347. doi: 10.3390/microorganisms11092347 (PMC10536989; doi:10.3390/microorganisms11092347)
Supplement: Supplementary file 1 [file microorganisms-11-02347-s001.zip › microorganisms-2577075-supplementary.pdf]

Supplementary Table S1. Patients' characteristics.

Characteristics	D614G period	Delta period	Total	*p*-value
Females, n (%)	13 (48.1%)	13 (61.9%)	26 (54.2%)	-
Age (years), median (IQR)	68 (IQR 61-72)	73 (IQR 63-82)	68 (IQR 62-76)	0.2127
Days from symptoms' onset to sampling, median (IQR)	21 (IQR 17-35)	21 (IQR 18-32)	21 (IQR 18-33)	0.5757
COVID-19 severity, n (moderate/severe)	9/18	8/13	17/31	-
Vaccine status, Not vaccinated	100% (27/27)	100% (21/21)	100% (48/48)	-
Period of infection	May–June 2020	October–November 2021		-

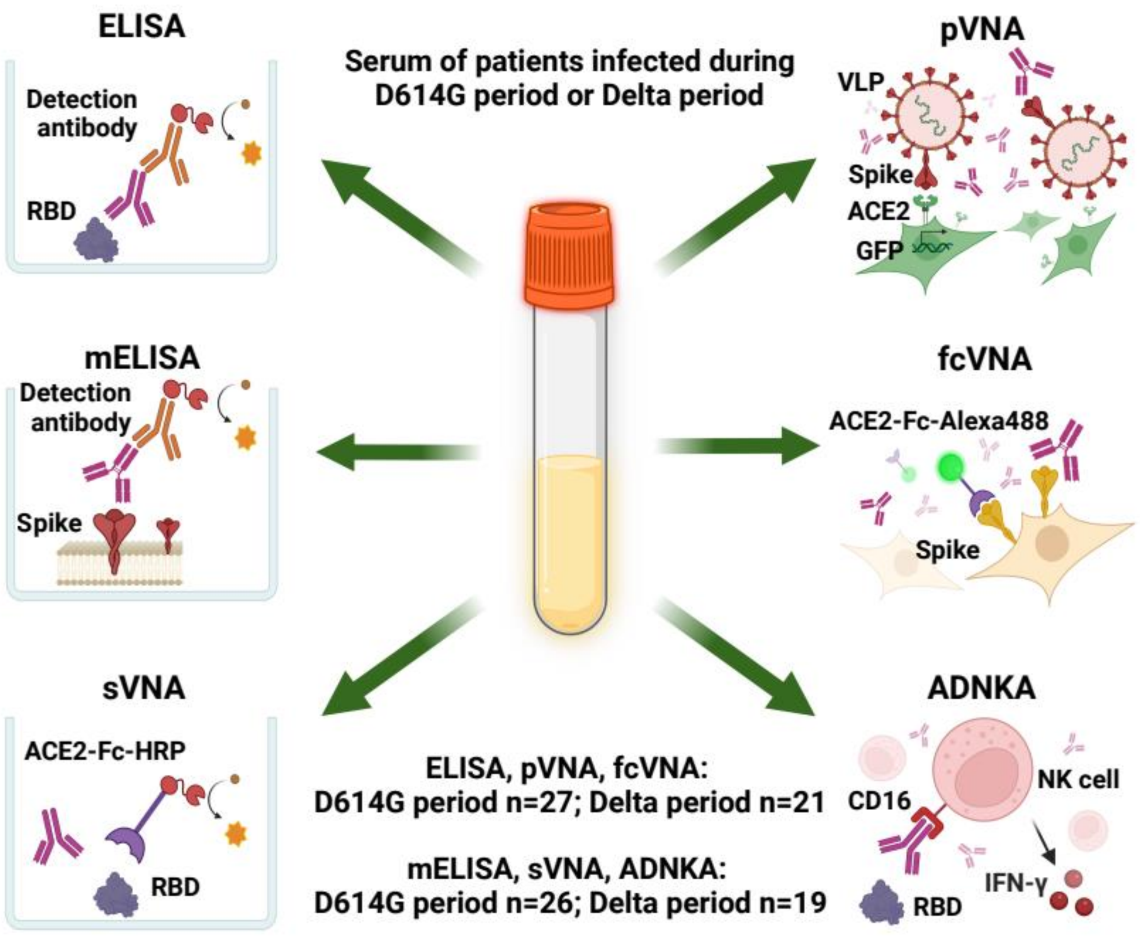

Supplementary Figure S1. Study design.
The study included two groups of patients hospitalized for acute COVID-19. Serum samples were collected from patients who had been infected during D614G (n = 27) or Delta (n = 21) periods. Sera were tested for SARS-CoV-2 binding and neutralizing antibodies with six assays based on standard ELISA, cell-membrane-based ELISA (mELISA), surrogate or pseudotyped virus-neutralization assay (sVNA or pVNA), flow-cytometry-based surrogate virus-neutralization assay (fcVNA) and antibody-dependent NK cell activation (ADNKA) with RBD and Spike protein from wild type, Delta and Omicron BA.1 variants as antigen.

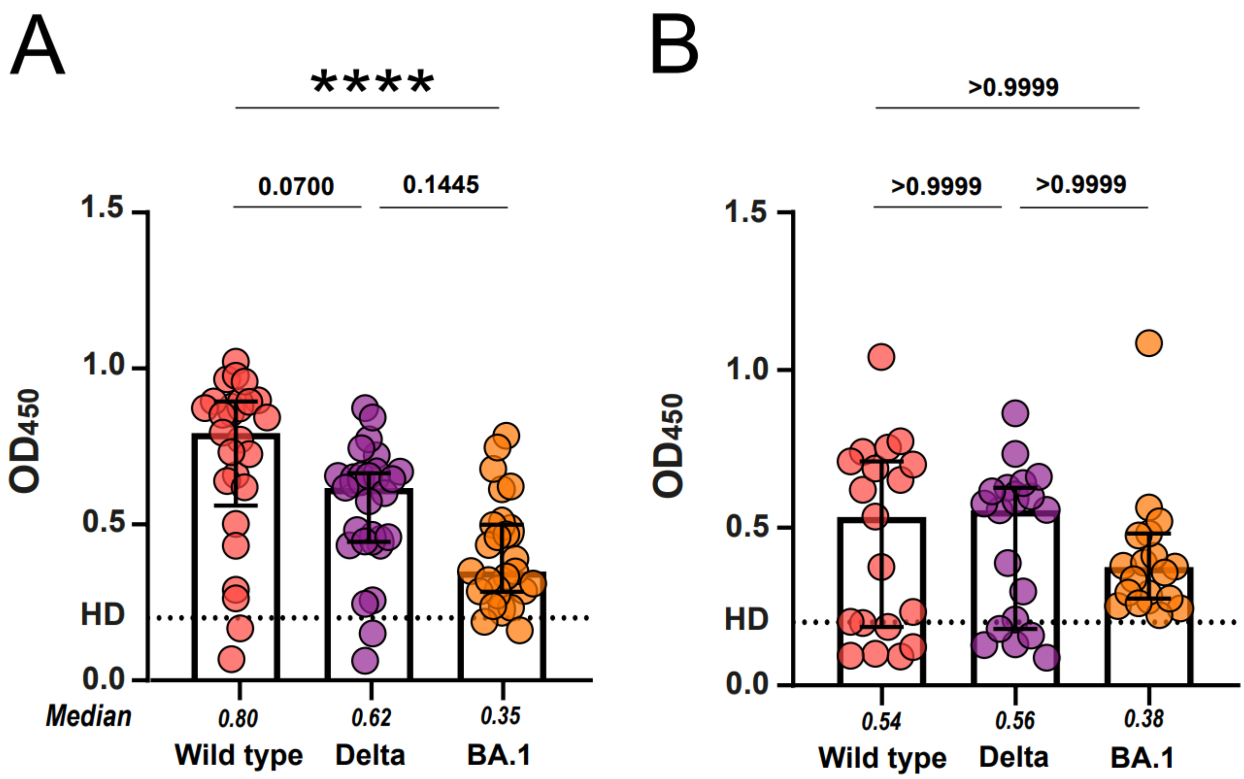

Supplementary Figure S2.
Reactivity of D614G (A) and Delta (B) period serum samples with WT, Delta, and BA.1 RBD in ELISA.

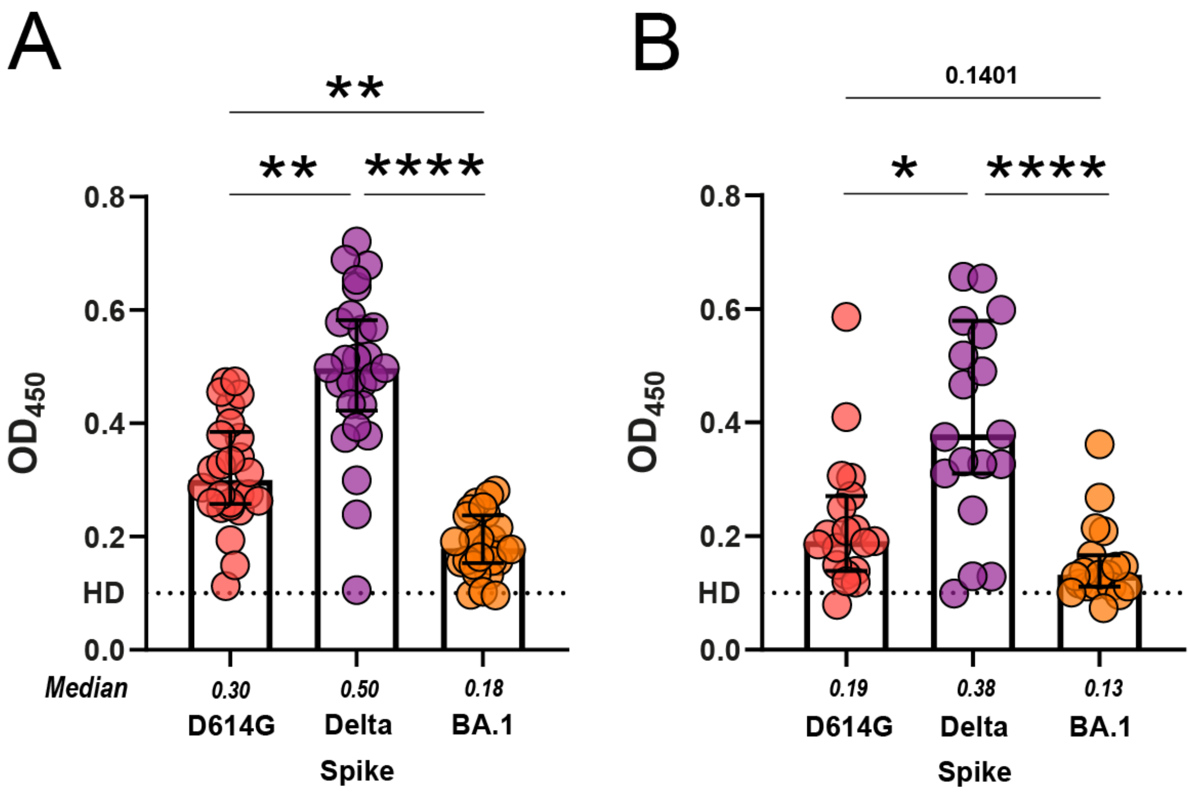

Supplementary Figure S3.
Reactivity of D614G (A) and Delta (B) period serum samples with D614G, Delta, and BA.1 S protein in membrane based ELISA (mELISA).

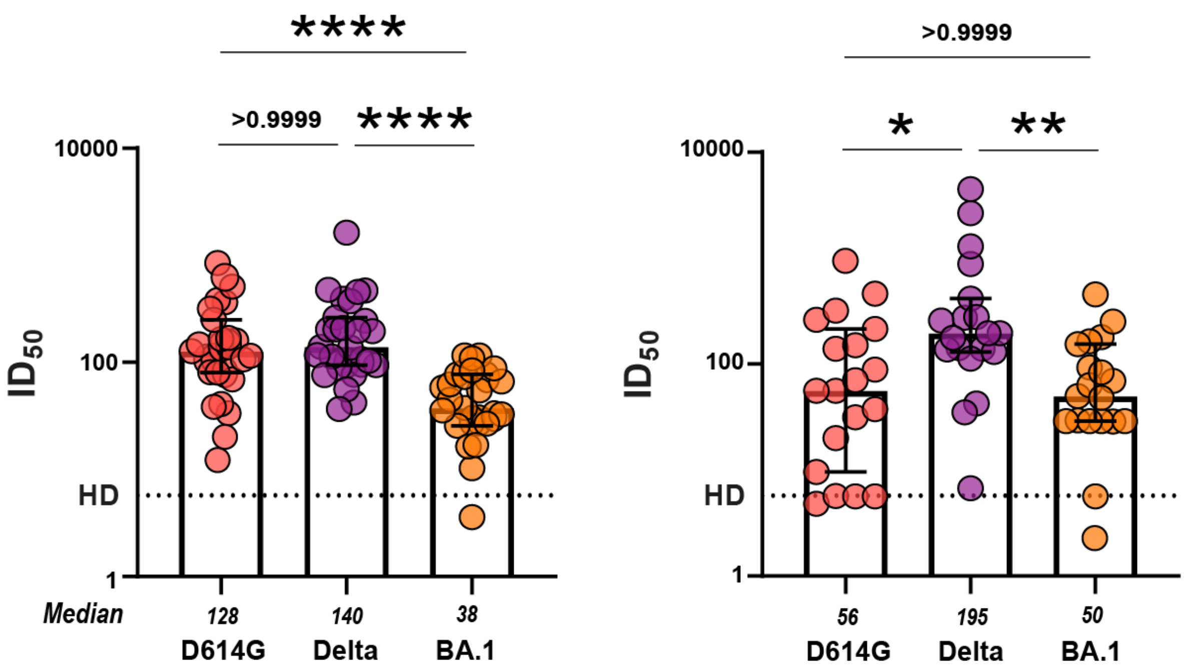

Supplementary Figure S4.
Neutralization antibody titers (ID_{50} values) against VLP pseudotyped with D614G, Delta, and BA.1 Spike variants in pseudotyped virus neutralization assay (pVNA) of D614G (left) and Delta (right) period serum samples.

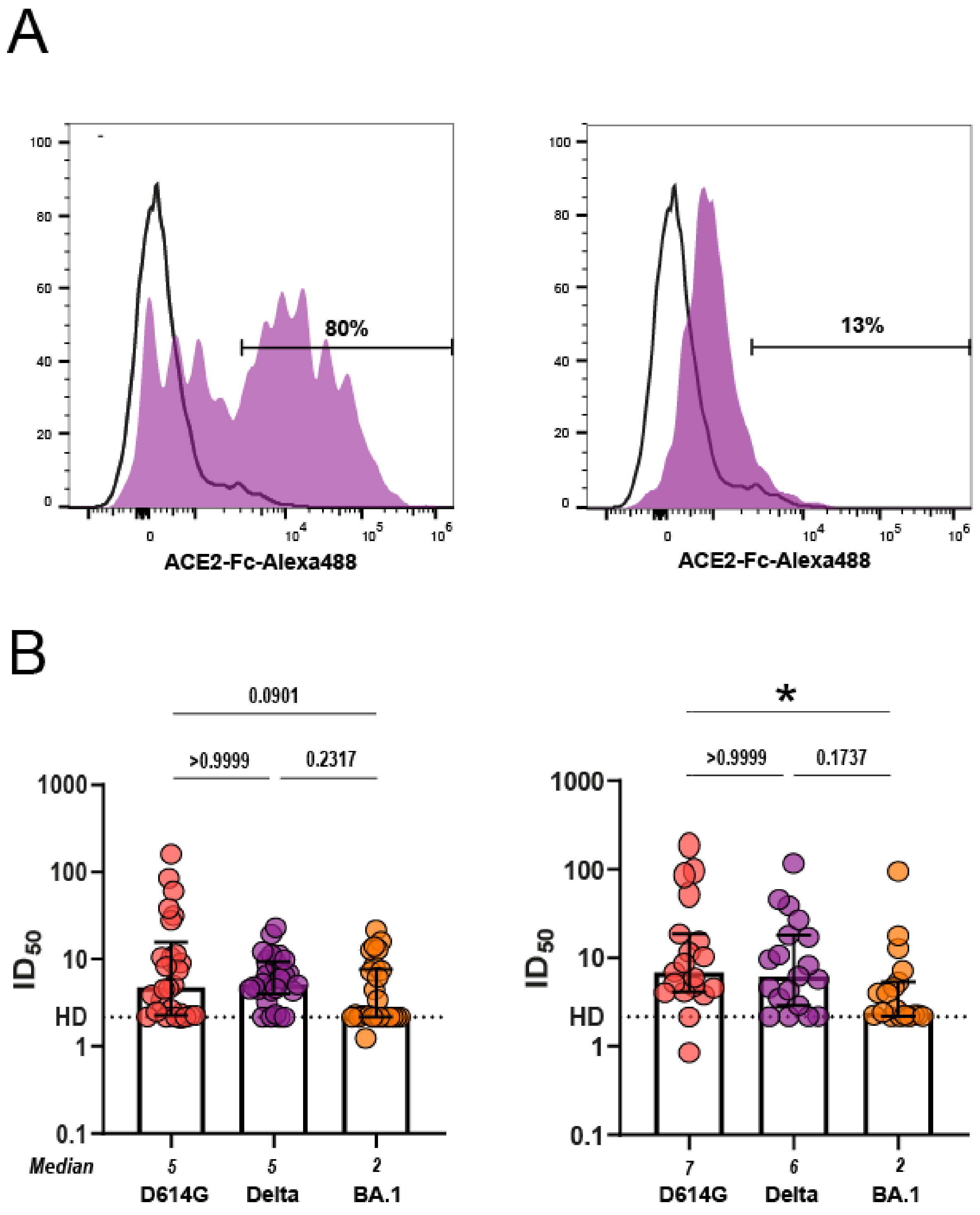

Supplementary Figure S5. Flow cytometry based surrogate virus neutralization assay (fcVNA).

A - representative flow plots for HEK293 cells transiently transfected with D614G Spike and stained with ACE2-Fc-Alexa Fluor 488 without addition of patient serum (left panel) or in the presence of patient serum (right panel). Untransfected cells stained with ACE2-Fc-Alexa Fluor 488 were used as a control (open plots). B - neutralization antibody titers (ID_{50} values) against D614G, Delta, and BA.1 Spike variants in fcVNA of D614G (left) and Delta (right) period serum samples.

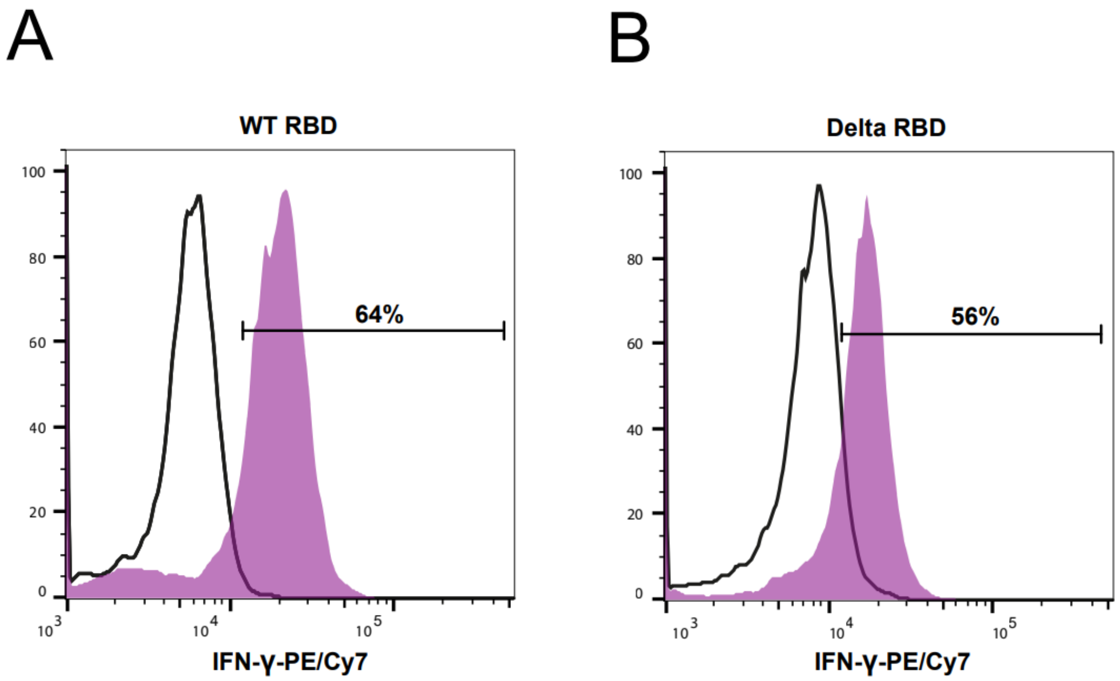

Supplementary Figure S6. Representative flow plots of antibody-dependent NK cell activation assay (ADNKA).

NK cells were incubated in wells coated with WT (A) or Delta (B) RBD, then permeabilized and stained with PE/Cy5-labeled antibody for IFN-γ. Numbers indicate the percentage of positive events. NK cells from uncoated wells stained with for IFN-γ-PE/Cy7 were used as a control (open plots).
